# Extreme low dose of 5-fluorouracil reverses MDR in cancer by sensitizing cancer associated fibroblasts and down-regulating P-gp

**DOI:** 10.1371/journal.pone.0180023

**Published:** 2017-06-29

**Authors:** Yan Ma, Yuhua Wang, Zhenghong Xu, Yongjun Wang, John K. Fallon, Feng Liu

**Affiliations:** 1 Division of Molecular Pharmaceutics, Center for Nanotechnology in Drug Delivery, Eshelman School of Pharmacy, University of North Carolina at Chapel Hill, Chapel Hill, North Carolina, United States; 2 School of Chinese Materia Medica, Guangzhou University of Chinese Medicine, Guangzhou, China; 3 School of Pharmacy, Shenyang Pharmaceutical University, Shenyang, China; University of Navarra, SPAIN

## Abstract

We conducted a prospective, meaningful study of extreme low dose of 5-fluorouracil (5FU) as a metronomic agent targeting cancer associated fibroblasts (CAFs) to reverse Multidrug resistance (MDR) by sensitizing cancer associated fibroblasts and down-regulating P-glycoprotein (P-gp). The combination of 5FU and Taxol inhibited resistant KB-8-5 tumor growth by 79% and H460/Tax-R tumor growth by 55%. The inhibition was significant for both tumor types compared with Taxol treatment alone (p<0.001 and p = 0.0067, respectively). Nevertheless, the low-dose 5FU (2.2 mg/kg compared to the therapeutic dose of 50–150 mg/kg) showed negligible tumor inhibitory effect. The tumor growth inhibition study on resistant tumors demonstrated that the continuous administration of low dose 5FU with Taxol significantly inhibited the tumor growth. The treatment overcomes drug resistance in tumors by down-regulating multi-drug resistance transporter protein (P-gp), and more importantly, by eliminating CAFs recruited by resistant tumors. Compared with traditional metronomic chemotherapy, 5FU as metronomic agent targeting CAFs can avoid the disadvantages resulted from the concomitant administration of antiangiogenetic drug. The approach has good translational potential for clinical trials when treating stroma-rich drug resistant tumors.

## Introduction

MDR is a major factor in the failure of many forms of chemotherapy [1–2]. Resistance to therapy has been correlated with the presence of at least one molecular ‘pump’ in tumor cell membranes, primarily P-gp, which actively expels chemotherapy drugs from the interior. While the third generation P-gp inhibitors are under development, there are no compounds currently available to ‘block’ P-gp mediated resistance in the clinic. This failure may be attributed to toxicity, adverse drug reactions and numerous pharmacokinetic issues [3]. Recently, it has been demonstrated that tumor stromal cells, such as CAFs, play important roles in MDR [4–6], not only promoting tumor progression but also inducing therapeutic resistance [5]. Therefore, targeting both CAFs and tumor cells using low toxicity agents could provide a novel approach and a potentially more effective treatment strategy for MDR in cancer.

Metronomic chemotherapy is defined as a chronic, uninterrupted, low dose administration of chemodrugs [7]. Its use surpassed that of conventional chemotherapy, where chemodrugs are administered in cycles of maximum tolerated dose (MTD), over a decade ago. It was initially to target genetically stable tumor endothelial cells, rather than drug resistant cancer cells [8]. However, results from numerous preclinical and clinical research studies on metronomic therapy revealed more putative mechanisms of actions, in addition to anti-angiogenesis, such as reversing the immunosuppressive tumor microenvironment [9] and promoting tumor dormancy.

Combined chemotherapy is used for the treatment of a number of malignancies. The incorporation of paclitaxel to 5FU and other agents regimen has shown an increase in response rates [10,11]. In spite of the different mechanisms of action and different toxicity profiles, the combination of paclitaxel/5FU can be administered safely [12,13]. In our previous studies, we found that the administration of paclitaxel plus low dose 5FU could effectively inhibit the tumor growth in the xenografts mouse model. Therefore, we hypothesized that the low dose 5FU can target tumor cells indirectly since it can affect the tumor microenvironments. Based on this, we evaluated the anti-cancer efficacy of a conventional, anti-proliferative chemodrug 5FU, as a metronomic agent combined with an anti-mitotic agent, paclitaxel.

## Materials and methods

### Materials

Paclitaxel injection (Taxol) was manufactured by Ben Venue laboratories, Inc. (Bedford, OH). Antibodies against P-gp, NF-κB, GAPDH horseradish peroxidase or fluorescence-conjugated anti-mouse or anti-rabbit whole IgG were obtained from Santa Cruz Biotechnology (San Diego, CA).

Resistant KB-8-5 cell line, sensitive KB-3-1 cell line (human mouth epidermal carcinoma cells) were obtained from National Cancer Institute. H460/Tax-R cell line (non-small lung carcinoma cells) was from Dr. Bingliang Fang from M.D. Anderson and NIH/3T3 fibroblast cells (mouse embryonic fibroblast cell line) were originally obtained from American Type Culture Collection (ATCC) (Manassas, VA). Cells were maintained in RPMI 1640 or DMEM medium (Life Technologies, Carlsbad, CA) containing 10% fetal bovine serum (Life Technologies), 100 unit/mL penicillin and 100 μg/mL streptomycin (Life Technologies). Cells were cultivated in a humidified incubator at 37°C and 5% CO_2_.

Nude mice were purchased from the National Cancer Institute (Bethesda, MD). All experiments performed on animals were in accordance with and approved by the Institutional Animal Care and Use Committee at the University of North Carolina at Chapel Hill.

### Cytotoxicity

KB-3-1, KB-8-5, H460, H460/Tax-R or NIH/3T3 fibroblast cells were seeded into 96-well plates at a density of 1× 10^4^ cells per well and allowed to adhere overnight. To determine the IC50 of 5FU for different cell lines, NIH/3T3, KB-8-5 and KB-3-1 cell were subject to 5FU treatment (from 0.1 μM to 50 μM). To determine the killing efficiency of combination therapy, various concentrations of PTX (from 0.1 nM to 100 nM) were added to the wells in the absence or presence of 5 μM 5FU for 48 h. Following incubation, 20 μl of MTT reagent (Sigma-Aldrich, St. Louis, MO) (5mg/ml in PBS) was added to the culture medium and the cells were incubated for an additional 4 h at 37°C. Then the culture media were carefully removed and 200 μl of DMSO were added to the wells to dissolve the formazan. Plates were read at 570 nm on a microplate reader using a Bio-Rad microplate imaging system (Hercules, CA) and results were expressed as cell viability (%) calculated as (OD of treated group / OD of control group) × 100.

### Western blot analysis and RT-PCR

KB-8-5 and H460/Tax-R resistant tumor cells were seeded into 10mm dishes and allowed to adhere overnight. 5 μM 5FU were added to the KB-8-5 and H460/Tax-R cells and incubated for 48 h. Following incubation adherent cells in culture dishes were washed with ice-cold PBS, lysed with RIPA buffer and scraped off the dish. The concentration of protein was quantified by Pierce BCA protein assay kit (Thermo Scientific Inc., Rockford, IL). Approximately 50 μg of protein from each sample was separated on NuPAGE 4–12% gradient SDS-PAGE (Life Technologies), and then transferred to polyvinylidene difluoride (PVDF) membrane (Bio-Rad, Hercules, CA). The membrane was blocked in 5% skim milk in PBS for 1 h. After incubation with primary antibody at 4°C overnight, PVDF membrane was washed with PBST (0.1% Tween 20 in PBS), and then incubated with secondary antibody for 1 h. Antibodies against P-gp, NF-κB and GAPDH were used at 1:2000 dilutions. An anti-mouse antibody conjugated with HRP at a dilution of 1:10,000 or an anti-rabbit IgG at a dilution of 1:2000 served as the secondary antibodies in the experiment. The specific protein bands were visualized using a chemiluminescence kit (Pierce, Rockford, IL). Chemiluminiscent signals were detected with the high-performance chemiluminescence film (GE Healthcare).

For RT-PCR analysis, H460/Tax-R and KB-8-5 cells were treated with the same condition as that for western blot analysis. After 48 h incubations, cells were harvested and total RNA were extracted using RNeasy mini kit (Qiagen, Venlo, Limburg). RNA was quantified and reverse transcribed using SuperScript^®^III reverse transcriptase (Life Technologies). The relative expression level of P-gp mRNA was determined using Taqman^®^ real time PCR system (Life Technologies) on ABI 7500 RT-PCR instrument (Life Technologies). The primers for P-gp (Mm00443188) and endogenous control GAPDH (Mm99999915_g1) were purchased from Life Technologies.

### Tumor growth inhibition assay

Anti*-*tumor activity was evaluated in KB-3-1, KB-8-5, H460, H460/Tax-R bearing athymic nude mice. Female nude mice (6–8 weeks) were used in all studies. Nude mice were subcutaneously inoculated with 5 × 10^6^ tumor cells into their right or left flanks to establish the xenograft model. Once the tumor mass in the xenograft was established, mice were randomly divided into corresponding groups (5 mice per group) and were injected with normal saline (the control group), PTX, 5FU or PTX+5FU. A drug dose of 5 mg/kg (calculated by PTX) was used for KB-3-1 and KB-8-5 tumor-bearing nude mice and 10mg/kg for H460, H460/Tax-R and NCI/ADR-Res tumor-bearing nude mice. The dose ratio of PTX to 5FU was 2.27:1 (w/w). Therapy was continued five times at every other day intervals via tail vein injections [14]. Tumor volumes were calculated as (length × width^2^)/2 from measurements taken every other day. Mice were sacrificed when the length of the tumor reached 2 cm. Furthermore, the toxicity of the formulations was determined by monitoring the animal behavior and the weight loss.

### Immunofluorescence and trichrome staining

Immunofluorescence detection of smooth muscle actin (SMA) expression in cancer associated fibroblasts were performed using paraffin sections prepared by UNC Tissue Procurement Core. The slides were deparaffinized and the antigen was retrieved by heating the samples at 90°C for 20 minutes in antigen retrieval buffer (Santa Cruz Biotechnologies). Rabbit anti-SMA antibody (Abcam Biotech, Cambridge, England) was incubated with sample at 1:100 dilution at 4°C overnight. Goat anti-rabbit IgG conjugated with Alexa Fluor 647 (Life Technologies) was used as secondary antibody at a dilution of 1:100. Images were taken using Nikon epi-fluorescence microscope (Nikon, Tokyo, Japan). TUNEL staining and trichrome staining and was performed according to the manufacturer’s instructions of TUNEL assay kit (Promega, Madison, WI) and Masson Trichrome staining kit (Sigma-Aldrich) respectively after deparaffinization of the tissue sections. Images were analyzed using ImageJ software and statistical analysis was conducted using GraphPad Prism.

### Tumor accumulation assay

The accumulation of Taxol in the tumor was evaluated by tracking ^3^H labeled Taxol. 5x10^6^ KB-8-5 cells were implanted in the athymic nude mice. When the tumor was established (~100 mm^3^), animals were intravenously injected 5FU at the dose of 2.22 mg/kg or PBS for three consecutive days. On the fourth day, the animals were intravenously injected with ^3^H labeled Taxol at the dose of 5mg/kg. The animals were sacrificed and the tumors were harvested for ^3^H paclitaxel analysis 24 h post-injection. Briefly, one hundred micrograms tumor tissue was diced well and dissolved in 1 ml tissue solubilizer (GE). Tissues were solubilized by incubating at 50°C overnight. Three hundred microliters solubilized samples were added to scintillation solution (Perkin Elmer, Waltham, Massachusetts) and examined using scintillation counter (BD, Franklin Lakes, NJ).

## Results and discussion

**Fig 1:**
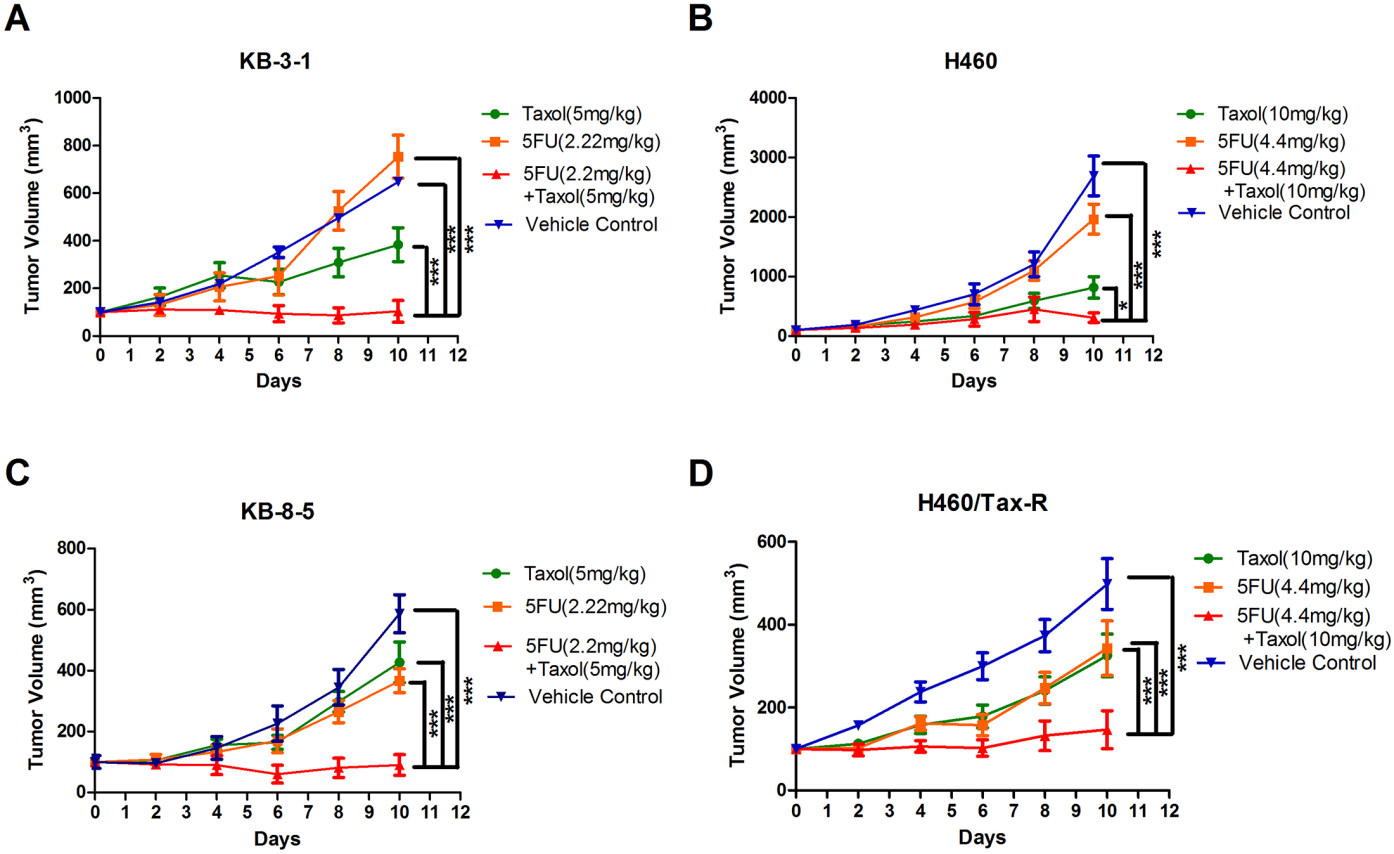
Inhibitory effects of low dose 5FU and Taxol on naïve and resistant tumor growth in a nude mouse model. When the tumors reached 100 mm^3^, animals received designated treatment every second day five times via tail vein injection. KB-3-1 (A) and KB-8-5 (B) tumors received 5mg/kg Taxol, while H460 (C) and H460/Tax-R (D) tumors received 10mg/kg Taxol. The ratio of paclitaxel to 5FU was 2.3:1 (w/w) for each treatment. Statistical analysis was performed using two-way ANOVA (*p<0.05; ***p<0.001).

The tumor growth inhibition study on KB-3-1 (human epidermoid carcinoma cell) bearing nude mice demonstrated that the continuous administration of 5FU at an extremely low dose (2.2 mg/kg compared to the therapeutic dose of 50–150 mg/kg) with Taxol (paclitaxel formulated in Cremophor EL) inhibited tumor growth by 73%. This was significant when compared with Taxol alone (*p*<0.0001) (Fig 1A). Nevertheless, the low-dose 5FU by itself showed negligible tumor inhibitory effect (*p* = 0.6980). The same trend was observed in the NCI-H460 (human lung carcinoma cell) tumor bearing mouse model, although the inhibition was not statistically significant (Fig 1B). As metronomic therapy is intended to prevent the acquired drug resistance by targeting genetically stable stromal cells, the exciting results led us to apply the low dose 5FU with Taxol combination to the treatment of resistant tumors. Two established resistant cell lines, KB-8-5 and H460/Tax-R, were used in the animal models. Compared with parental cell lines, KB-8–5 cells overexpress P-gp thus employing reduction of cytosolic drug accumulation as a multidrug resistance mechanism [15], while H460/Tax-R cells overexpress both P-gp and α-tubulin thus counteracting the effect of paclitaxel [16]. The low dose administration of 5FU effectively overcame drug resistance and enhanced the drug response in both Taxol resistant tumor bearing mouse models (Fig 1C and 1D). The combination of 5FU and Taxol inhibited KB-8-5 tumor growth by 79% and H460/Tax-R tumor growth by 55%. The inhibition, when compared with Taxol treatment alone was significant for both tumor types (*p*<0.001 and *p* = 0.0067, respectively) (Fig 1C and 1D). Particularly for KB-8-5 bearing tumors, the combined therapy led to partial remission at the endpoint of the treatment. On the contrary, treatment with Taxol or 5FU alone induced little drug response. Therefore, low dose 5FU seemed to reverse resistance status of the tumors, making them vulnerable to treatment with Taxol.

**Fig 2:**
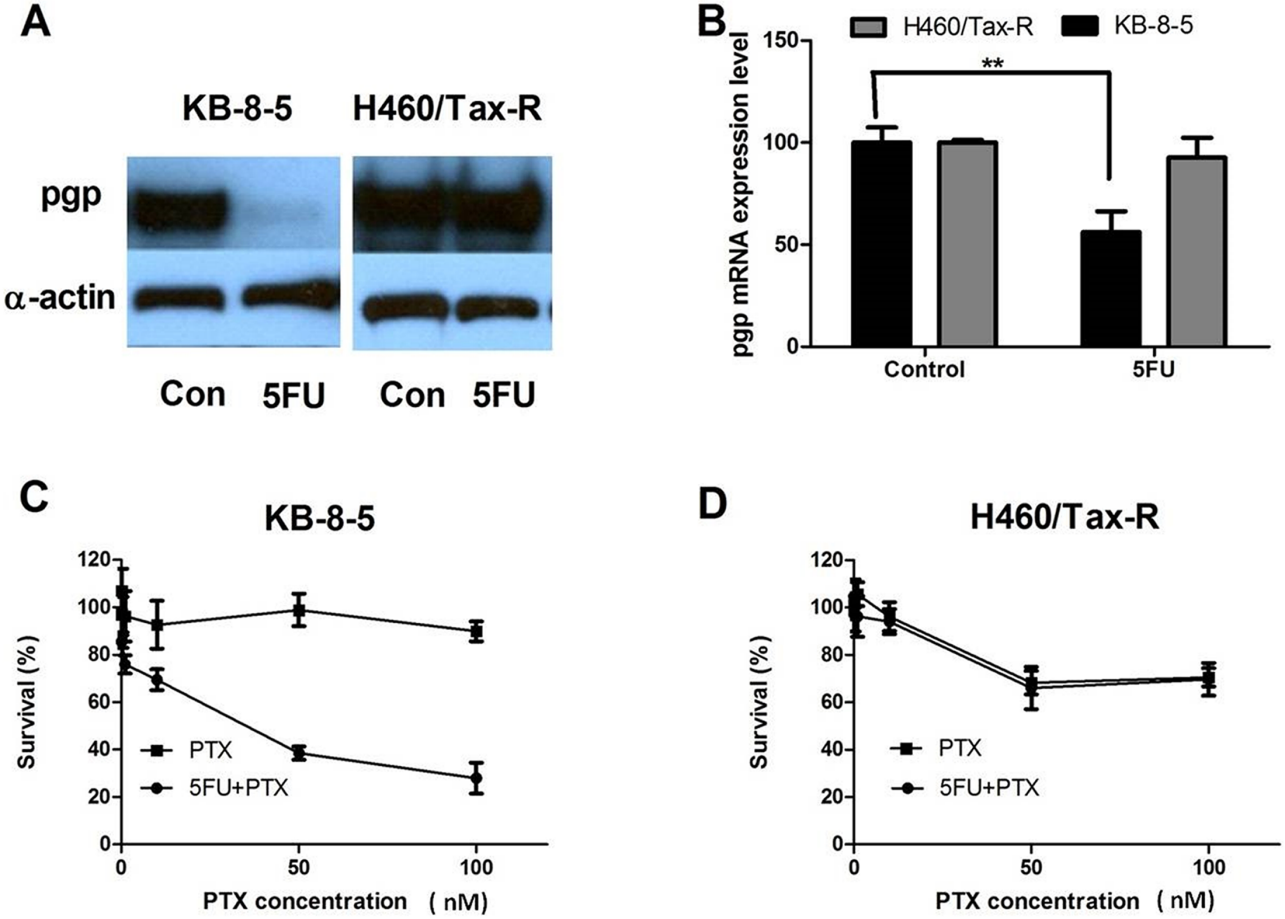
*In vitro* investigations of mechanisms of action for the reversal of drug resistance in KB-8-5 and H460/Tax-R cell lines. The overexpression of Pgp was inhibited in KB-8-5 cells but not H460/Tax-R cells after exposure to 5 μM 5FU (A). RT-PCR analysis showed Pgp mRNA levels to be consistent with protein levels (B). MTT assay demonstrated that low dose of 5FU (5 μM) reversed the resistance status of KB-8-5 cells to the treatment of Taxol (PTX) (C). However, no difference was observed in the H460/Tax-R cell line (D). Statistical analysis was performed using Student’s *t*-test (** *p*<0.01).

The *in vivo* results led us to investigate the mechanisms underlying the reversal of drug resistance status *in vitro*. The P-gp expression levels of both resistant cell lines after exposure to low dose 5FU was examined by Western blot. A significant down-regulation of P-gp was observed in the 5FU treated KB-8-5 cell line, whereas no change was observed in H460/Tax-R cell line (Fig 2A). The same trend was also observed in RT-PCR analysis, indicating that the down-regulation was modulated at the transcriptional level (Fig 2B). The inhibition of P-gp expression correlated with the chemo-sensitivity of the cell lines, reflected by the results of the MTT assay (Fig 2C and 2D). The combination treatment only showed mildly inhibitory effects in the H460/Tax-R cell line (Fig 2D). The drug response *in vitro* was not consistent with the effects *in vivo*. Therefore, it is speculated that the therapeutic target of low dose 5FU is more than just the cancer cells. It is possible that stromal cells in the tumor microenvironment *in vivo* could be affected thus changing the drug resistance status.

**Fig 3:**
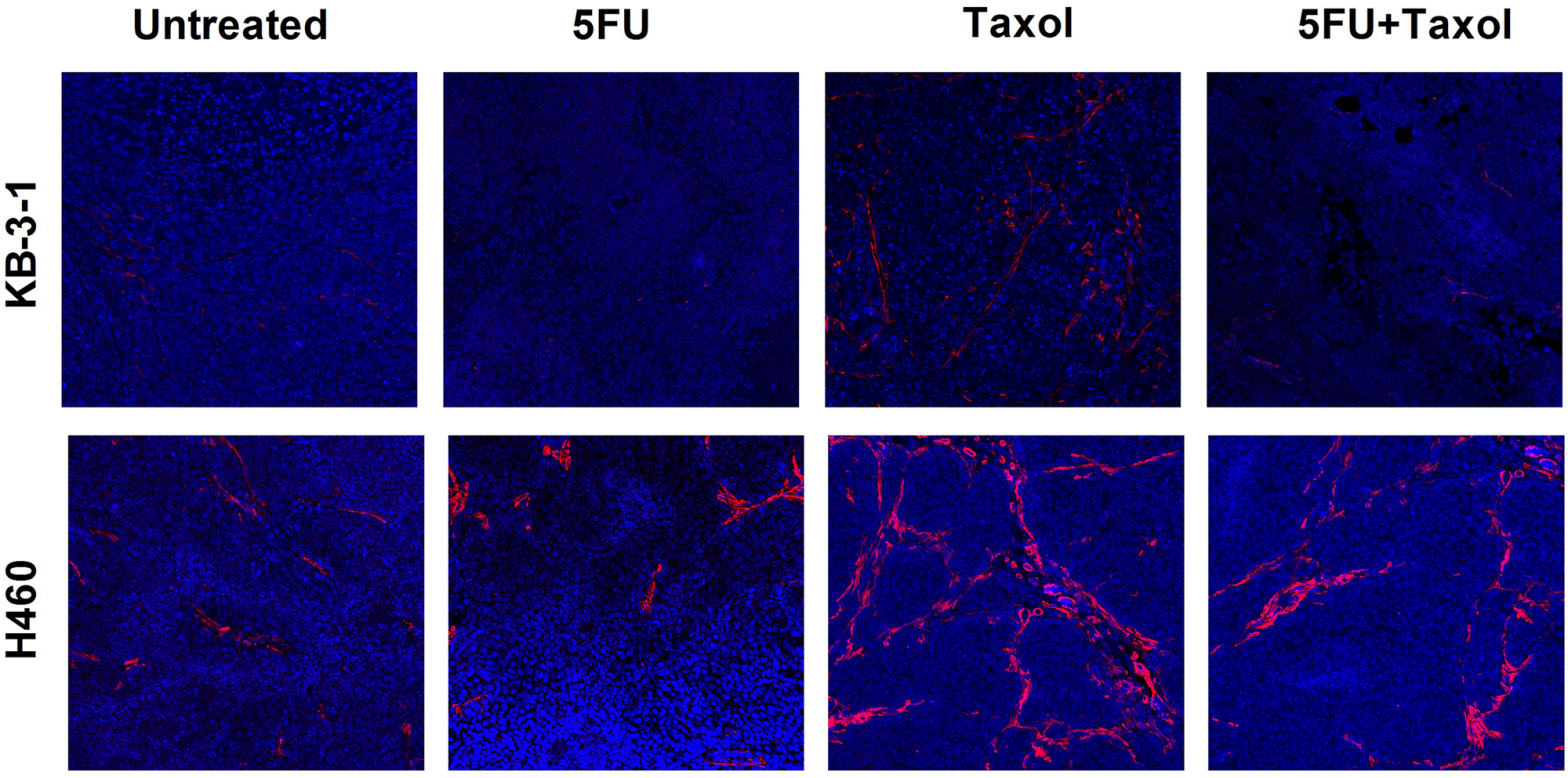
Immunofluorescent staining of α-SMA on naive tumor (KB-3-1 and H460) harvested at the end point of the tumor inhibition studies (Fig 1A and 1B).

**Fig 4:**
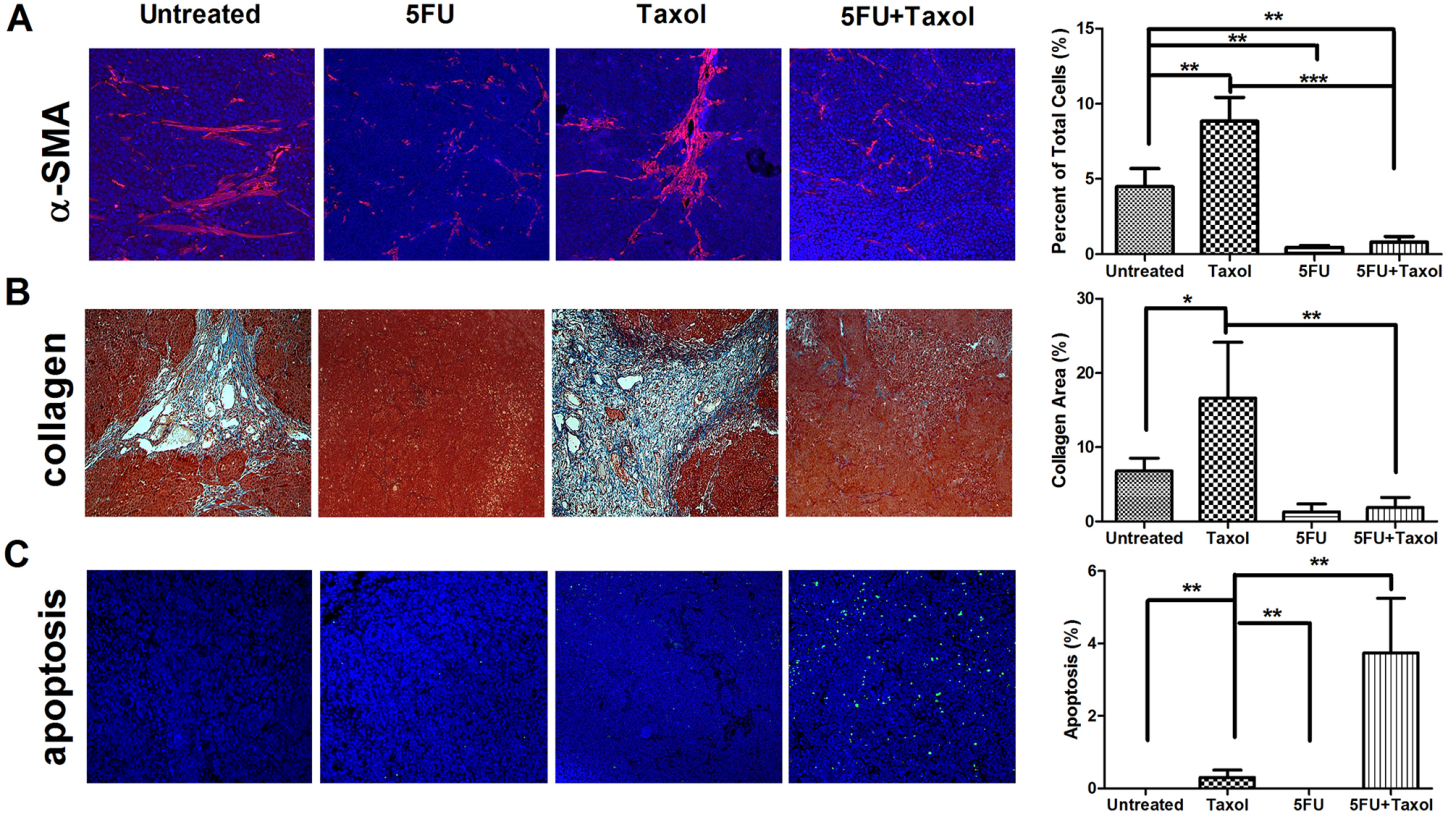
Immunofluorescent and histological analysis of KB-8-5 tumor tissue after treatment. Approximately 4.5% of cells in KB-8-5 tumors are CAFs and the Taxol treatment further increased the recruitment of CAFs up to 8.8%. 5FU, however, could reduce the percent of CAFs to 0.9% after the combination therapy (A). Increased percentage of CAFs in the tumor led to increased expression of collagen, which could be reversed by elimination of CAFs using 5FU (B). Elimination of CAFs contributed to the increased number of apoptotic cells in the drug resistant tumors (C). Statistical analysis was performed using the Student’s *t*-test (* *p*<0.05, ** *p*<0.01, *** *p*<0.001).

**Fig 5:**
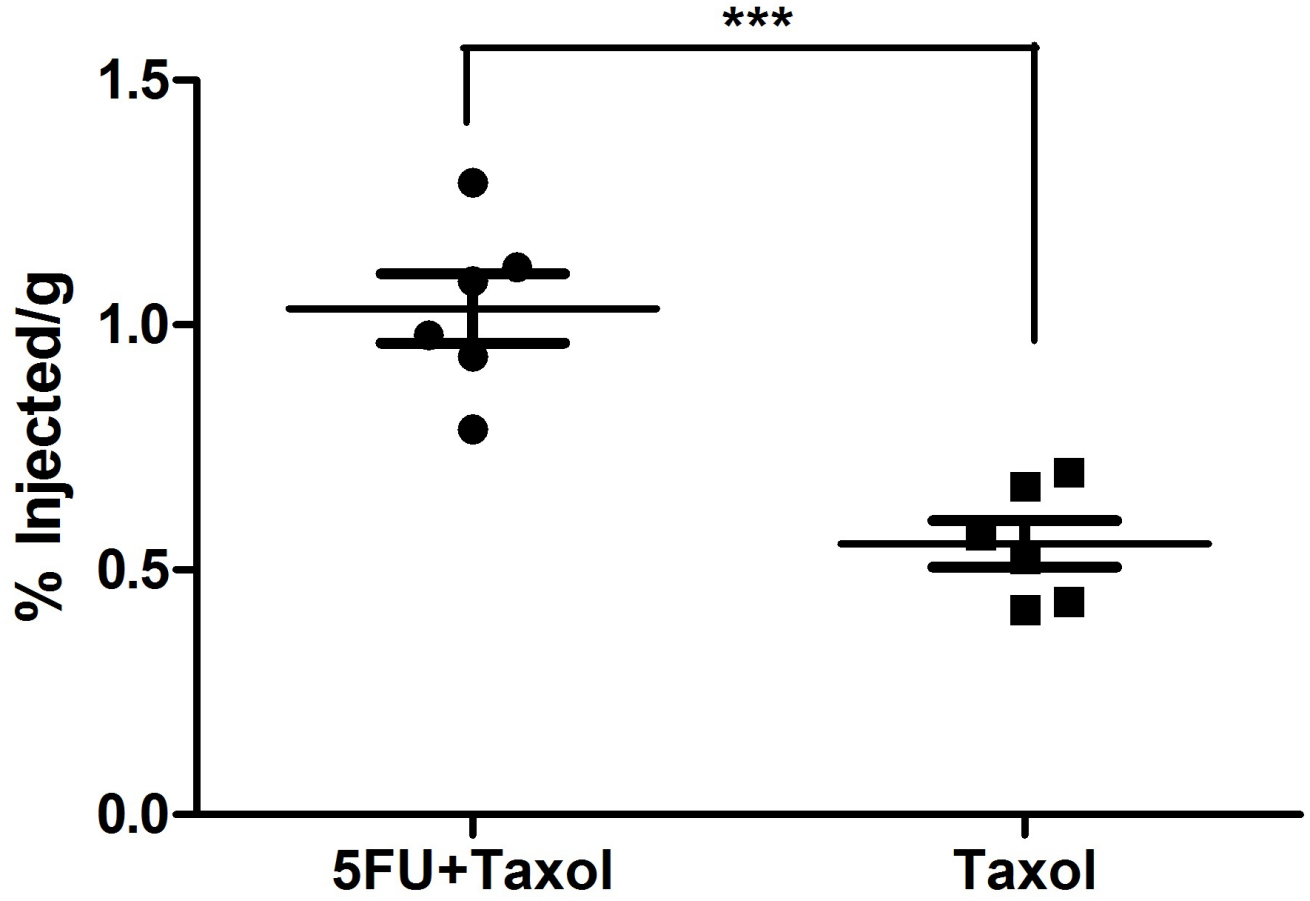
^3^H labelled Taxol accumulation in the KB-8-5 tumors after the tumor bearing mice received three injections of 5FU+Taxol or Taxol alone.

CAFs support tumor growth via modulation of the extracellular matrix, secretion of growth factors, suppression of immunosurveillance mechanisms and alteration of tumor metabolism. There is growing evidence that CAFs sustain the stemness of cancer cells, stemness being involved in cancer cell drug resistance mechanisms [17–19]. We therefore explored the status of CAFs after treatment using immunofluorescence staining of α-smooth muscle actin (α-SMA), a marker for CAFs. The results demonstrated that treatment of naive KB-3-1 and H460 tumors with Taxol induced extensive infiltration of CAFs into the tumor microenvironment (Fig 3), the infiltration being greater than for untreated tumors (Fig 3). This suggested a tendency to acquire drug resistance which was reflected by constant growth rate at the late stage of the treatment (Fig 1A and 1B). In the resistant tumors (KB-8-5 and H460/Tax-R), an extensive infiltration of CAFs was observed in the untreated groups (Fig 4A) with the infiltration being increased after the treatment of Taxol alone. All of these results combined suggest that infiltration of CAFs is correlated with drug resistance status *in vivo*. Low dose administration of 5FU eliminated the CAFs possibly due to higher vulnerability of the fibroblasts to so-called anti-fibrotic agents (Fig 4A). In KB-8-5 cells, 5FU treatment could down-regulate P-gp expression (Fig 2A), sensitizing the cancer cells to Taxol treatment. Meanwhile, increased percentage of CAFs in the tumor led to increased expression of collagen, which could be reversed by elimination of CAFs using 5FU (Fig 4B). The elimination of extracellular matrix reduced the interstitial fluid potential and increased the drug accumulation of Taxol by approximately two-fold (0.55±0.12% *vs* 1.03±0.17% injected dose/gram) (Fig 5). Elimination of CAFs also contributed to the increased number of apoptotic cells in the drug resistant tumors (Fig 4C). Reversal of cellular resistance and increase of drug accumulation caused by low dose 5FU resulted in 10-times more apoptotic cells in the KB-8-5 tumor following Taxol treatment than when Taxol treatment alone was used (3.73±1.51% *vs* 0.30±0.20%).

**Fig 6:**
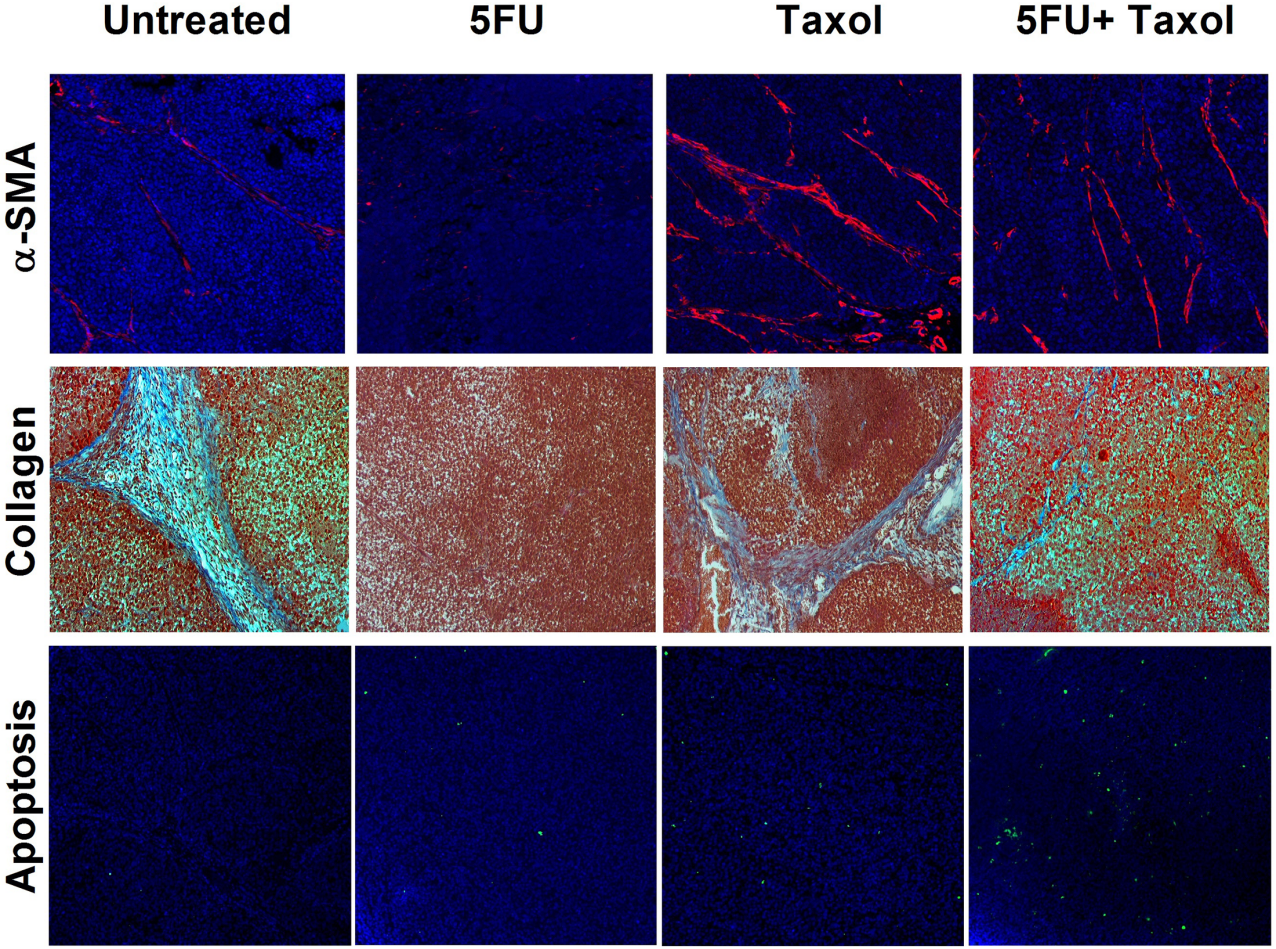
Immunofluorescent and histological analysis of H460/Tax-R tumor tissue after treatment. CAFs were observed in untreated H460/Tax-R tumor and the percentage of CAFs increased after treatment with Taxol. 5FU, however, could reduce the percentage of CAFs. Increased presence of CAFs in the tumor led to increased expression of collagen, which could be reversed by elimination of CAFs using 5FU.

When the groups were treated with 5FU alone or 5FU+Taxol, the percentage of CAFs in the resistant tumor was decreased as well as the decreased expression of collagen was also detected (Fig 6). Elimination of CAFs also contributed to the increased number of apoptotic cells in the H460/Tax-R resistant tumors (Fig 6). It is noteworthy that H460/Tax-R cells showed exactly the same pattern with respect to CAFs as KB-8-5 cells, although 5FU failed to down-regulate the P-gp expression level *in vitro* (Fig 2B). Elimination of CAFs seemingly prevents the development of resistance and reverses the status of resistant cancer cells. Therefore, it is highly likely that CAFs recruited by the resistant tumor are required to maintain the resistance status of the cells in the absence of drug treatment. The removal of the CAFs thus disrupted this conditioning microenvironment and sensitized the resistant cells to the treatment.

## Conclusions

This is one of the first studies that has demonstrated extremely low dose of 5FU as a metronomic agent targeting CAFs and reversing tumor MDR. P-gp determination and α-SMA immunofluorescence staining clarified the relationship between P-gp expression, fibroblast levels and tumorigenesis. In our previous study, we had found that 5FU had no influence on the tumor cells at the chosen concentrations *in vitro*, but the sensitivity enhancement was obviously observed when the NIH/3T3 fibroblast cells were treated with 5FU at the same concentration. Therefore, we think that 5FU play an important role in the elimination of CAFs in resistant tumors. CAFs is the structural and chemical support during tumor progression, once failure to recruit fibroblasts *in vivo* for tumor cells containing high levels of P-gp lies in less structural and chemical support and more favors antitumor agents kill the resistant tumor cells.

In a word, the treatment overcomes drug resistance in tumors not only by down-regulating multi-drug resistance transporter protein (P-gp) but also more importantly by eliminating CAFs recruited in resistant tumors. The final promotion of drug accumulation inside the tumor is the combination of the first two. Classically, metronomic chemotherapy is thought to exert its anticancer activity mainly by inhibiting tumor angiogenesis. In the study, this mechanism of action for metronic therapy has not been previously reported to the best of our knowledge. Furthermore, metronomic agent targeting CAFs enable to avoid the disadvantages caused by the concomitant administration of antiangiogenetic drugs. The results also suggest that CAFs not only contribute to the acquisition of drug resistance, and more pivotal to the maintenance of cellular resistance. Essentially, the approach has good translational potential for clinical trials when treating stroma-rich drug resistant tumors.
